# Clostridioides difficile S-Layer Protein A (SlpA) Serves as a General Phage Receptor

**DOI:** 10.1128/spectrum.03894-22

**Published:** 2023-02-15

**Authors:** Alexia L. M. Royer, Andrew A. Umansky, Marie-Maude Allen, Julian R. Garneau, Maicol Ospina-Bedoya, Joseph A. Kirk, Gregory Govoni, Robert P. Fagan, Olga Soutourina, Louis-Charles Fortier

**Affiliations:** a Department of Microbiology and Infectious Diseases, Faculty of Medicine and Health Sciences, Université de Sherbrooke, Sherbrooke, Québec, Canada; b Université Paris-Saclay, CEA, CNRS, Institute for Integrative Biology of the Cell (I2BC), Gif-sur-Yvette, France; c Molecular Microbiology, School of Biosciences, University of Sheffield, Sheffield, United Kingdom; d AvidBiotics, South San Francisco, California, USA; Wuhan University

**Keywords:** *Clostridioides difficile*, phage receptor, phage-host interactions, S-layer, SlpA, bacteriophage therapy, bacteriophages

## Abstract

Therapeutic bacteriophages (phages) are being considered as alternatives in the fight against Clostridioides difficile infections. To be efficient, phages should have a wide host range, buthe lack of knowledge about the cell receptor used by C. difficile phages hampers the rational design of phage cocktails. Recent reports suggested that the C. difficile surface layer protein A (SlpA) is an important phage receptor, but available data are still limited. Here, using the epidemic R20291 strain and its FM2.5 mutant derivative lacking a functional S-layer, we show that the absence of SlpA renders cells completely resistant to infection by ϕCD38-2, ϕCD111, and ϕCD146, which normally infect the parental strain. Complementation with 12 different S-layer cassette types (SLCTs) expressed from a plasmid revealed that SLCT-6 also allowed infection by ϕCD111 and SLCT-11 enabled infection by ϕCD38-2 and ϕCD146. Of note, the expression of SLCT-1, -6, -8, -9, -10, or -12 conferred susceptibility to infection by 5 myophages that normally do not infect the R20291 strain. Also, deletion of the D2 domain within the low-molecular-weight fragment of SlpA was found to abolish infection by ϕCD38-2 and ϕCD146 but not ϕCD111. Altogether, our data suggest that many phages use SlpA as their receptor and, most importantly, that both siphophages and myophages target SlpA despite major differences in their tail structures. Our study therefore represents an important step in understanding the interactions between C. difficile and its phages.

**IMPORTANCE** Phage therapy represents an interesting alternative to treat Clostridioides difficile infections because, contrary to antibiotics, most phages are highly species specific, thereby sparing the beneficial gut microbes that protect from infection. However, currently available phages against C. difficile have a narrow host range and target members from only one or a few PCR ribotypes. Without a clear comprehension of the factors that define host specificity, and in particular the host receptor recognized by phages, it is hard to develop therapeutic cocktails in a rational manner. In our study, we provide clear and unambiguous experimental evidence that SlpA is a common receptor used by many siphophages and myophages. Although work is still needed to define how a particular phage receptor-binding protein binds to a specific SLCT, the identification of SlpA as a common receptor is a major keystone that will facilitate the rational design of therapeutic phage cocktails against clinically important strains.

## INTRODUCTION

With the increasing antibiotic resistance worldwide, there is a regained interest in phage therapy nowadays (1). Bacteriophages (or phages) have the advantage of being highly specific toward their bacterial host, thereby sparing surrounding bacterial species. Since broad-spectrum antibiotics often lead to collateral damage, more targeted therapeutics are needed, especially in the context of gastrointestinal infections (2). Clostridioides difficile is one of the Gram-positive pathogens for which phage therapy has been proposed as a potential therapeutic alternative (3, 4). C. difficile is the main cause of antibiotic-associated diarrhea in hospitals, and therapeutic solutions are limited, particularly in the context of recurrent infections (2). C. difficile takes advantage of the microbiota dysbiosis caused by broad-spectrum antibiotics to colonize and persist in the gastrointestinal tract. This opportunistic bacterium induces severe and often recurrent intestinal infections driven by the production of toxins (TcdA and TcdB). Since the emergence of the ribotype 027 epidemic strain, C. difficile is considered an urgent threat by the U.S. Centers for Disease Control and Prevention (5, 6).

C. difficile is susceptible to infection by two structurally different types of phages; more specifically, siphophages, which possess long, flexible and noncontractile tails, and myophages, which possess nonflexible and contractile tails. Like many other phages, those infecting C. difficile generally have a narrow host range (3, 7, 8), which can be the result of multiple factors, including the presence of an endogenous clustered regularly interspaced short palindromic repeat (CRISPR) system (9), restriction-modification systems (10), phage superinfection exclusion mechanisms, such as CwpV (11), and repressor-mediated resistance provided by endogenous prophages (12, 13). Another reason that can explain a narrow host range is the absence of a suitable host receptor at the surface of the target bacteria. The study of phage receptors is therefore crucial both to better select suitable therapeutic phages and because phage resistance often comes from mutation of the receptor (13).

Recognition and binding to a specific receptor at the surface of a bacterial cell is the first and key step for a successful bacteriophage infection. The adsorption process involves close interaction between a cell surface component and a phage counterpart, generally located at the tip of the tail in the case of tailed phages. This adsorption generally occurs in two steps: the first one involves reversible binding of the phage to the host cell surface through tail fibers or other decorations, and the second one is the irreversible binding of the phage receptor-binding protein (RBP) to the same host receptor, a different one, or both (7, 14).

Substantial structural work has been done on model phages like the myophages T4 (15) and A511 (16), as well as the siphophages λ (17), SPP1 (18), and p2 (19, 20). This allowed the identification of key tail components involved in receptor recognition, including baseplate components, tail fibers, and the RBP, which plays a central role in receptor recognition (21). Because phage genomes are highly modular and genomic synteny is generally observed, it is now possible to predict with some confidence the RBP and other tail components that compose the host recognition machinery by homology searches in public repositories. The identification of phage host receptors is more complex, however. The isolation of a spontaneous bacteriophage-insensitive mutant (BIM) is one of the best ways to identify such receptors (22, 23). A large diversity of phage receptors has been characterized to date, revealing bacterial evolutionary strategies to overcome phage infection. Such receptors include proteins, polysaccharides, lipopolysaccharides, capsules, pili, and flagella (24). Much work was done in Gram-negative bacteria, and less is known about phage receptors in Gram-positive bacteria (21). Due to the constant arms races between phages and bacteria, there is a large variability among phage receptors and RBPs, and as such, phage-host interactions remain poorly understood for most bacterial species (25, 26). In C. difficile, the identity of phage receptors was unknown until recently, and current data point to the cell surface protein SlpA as being an important phage receptor.

The C. difficile cell surface is composed of a dense proteinaceous array, called the surface layer or “S-layer,” which contains various cell wall-associated proteins, among which SlpA is the most abundant (27). The S-layer was shown to play various roles in cell adhesion and pathogenesis, immunity, permeability of the bacterial cell, and motility (28–30). The SlpA protein is posttranslationally cleaved into two fragments by the cell wall-associated protease Cwp84 and secreted at the cell surface (27). A high-molecular-weight (HMW) moiety is attached to the cell wall through cell wall binding domains, while a second, low-molecular-weight (LMW) fragment is reassociated with the HMW (31). To date, 14 different SlpA isoforms (also named S-layer cassette types [SLCTs]) have been described where the HMW part of the protein is the most conserved and the LMW portion, which is the most exposed at the bacterial surface, is the most variable (32, 33).

Diffocins are phage tail-like R-type bacteriocins structurally resembling myophage tails (34). A recent study revealed that diffocins engineered with a myophage-derived RBP called Avidocin-CD291.2 attach to and lyse C. difficile cells upon binding to SlpA, which acts as their main receptor (35). While studying Avidocin-CD291.2, complete resistance was observed in two spontaneous mutants of the C. difficile epidemic strain R20291 (ribotype 027). These mutant strains, FM2.5 and FM2.6, carry point mutations that either introduce a premature stop codon or cause a translational frameshift, leading to severe truncation of SlpA. Complementation of the FM2.5 mutant with different SLCTs carried on plasmids rescued susceptibility to killing by different Avidocin-CDs (35). Genetic engineering of these Avidocin-CDs by replacement of their RBP with a predicted prophage RBP conferred the prophage’s host range on the Avidocin-CD, which led the authors to suggest that SlpA is likely the receptor used by phages as well (35, 36). Another study, based on gel retardation assays with S-layer extracts, also suggested that the S-layer was targeted by the C. difficile phage ϕHN10 (37). Whittle et al. showed that introduction into strain 630 of plasmids carrying either SLCT-6 or SLCT-H2/6 allowed adsorption of phage ϕCD1801, a myophage that targets ribotype 078 isolates but does not infect strain 630 (ribotype 012) (38). Finally, another recent report suggests that ϕCDHS-1, a siphophage infecting the R20291 strain, also recognizes SlpA, because it could not infect the FM2.5 mutant (39). Altogether, these data support the idea that SlpA is a receptor for certain phages, but in all the above-mentioned studies, the evidence available is either indirect or incomplete.

In this study, we sought to fill this knowledge gap by providing an unambiguous experimental demonstration that SlpA is a general phage receptor in C. difficile. To achieve this, we used the R20291 FM2.5 *slpA* mutant strain complemented with 12 different SlpA isoforms and a collection of 8 different phages. Productive infections and adsorption assays allowed us to clearly demonstrate that SlpA is a general receptor used by many phages to infect C. difficile. The data described herein provide a solid foundation for future work aiming at better characterizing phage-host interactions in C. difficile.

## RESULTS

### Loss of SlpA confers resistance to phage infection.

Recent studies pointed toward a role of the C. difficile surface layer protein SlpA in phage infection (35, 37, 38, 40). However, in some cases, only indirect evidence based on engineered Avidocin-CD (35) or adsorption assays alone (37, 38) are currently available. Furthermore, previous studies suggesting that the S-layer or SlpA is a phage receptor were conducted with myophages only. Hence, whether SlpA also serves as a receptor for siphophages needs to be demonstrated. This question is of great importance since the tail architectures of myophages and siphophages are very different and there is no indication that both phage families use the same type of receptor to infect their host.

We have previously shown that the wild-type (WT) R20291 epidemic strain is susceptible to infection by the siphophages ϕCD38-2, ϕCD111, and ϕCD146 (8). To determine whether SlpA could also serve as a receptor for siphophages, we tested the susceptibility of the FM2.5 *slpA* mutant strain described by Kirk et al. (35) to infection by these three well-described phages.

**FIG 1:**
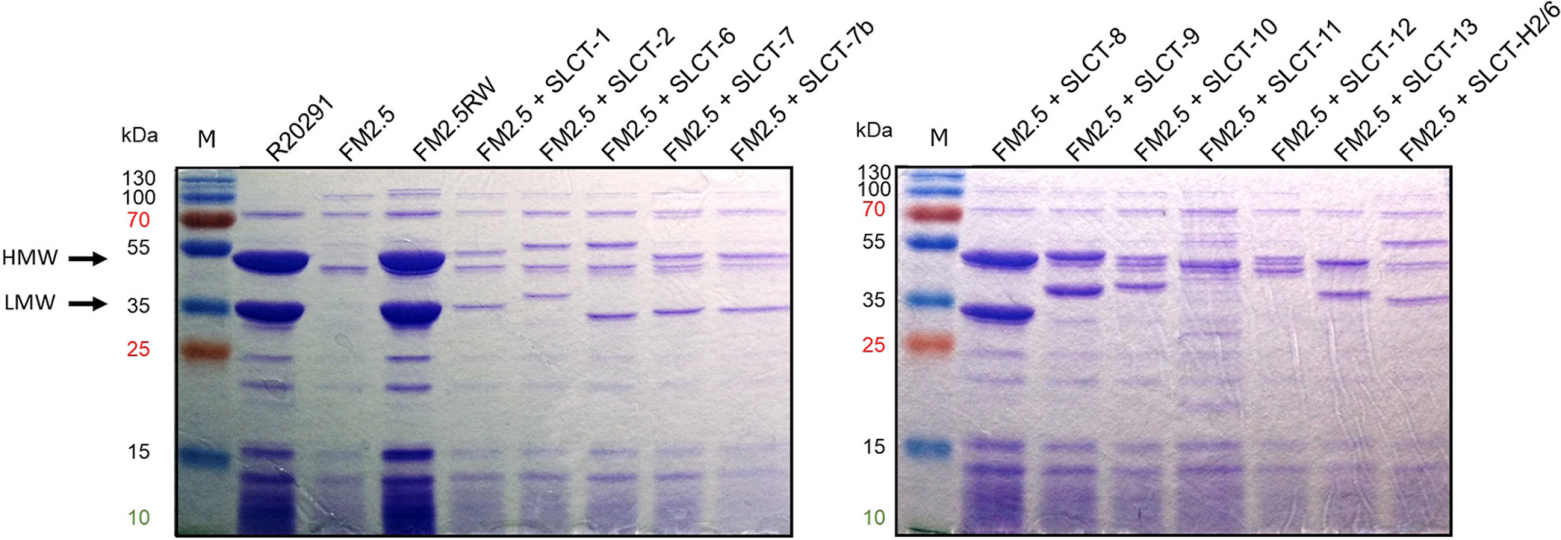
Coomassie-stained 12% SDS-PAGE gels showing glycine extractions of surface proteins from WT R20291, the FM2.5 *slpA* mutant, the FM2.5RW watermarked revertant, and the FM2.5 *slpA* mutant complemented with a plasmid encoding one of the 12 other SLCTs tested. The expression of the SLCTs was under the control of the P_tet_ promoter, and induction was performed with 20 ng/mL anhydrotetracycline. The arrows indicate the two major bands corresponding to the HMW and LMW units of the SLCT-4 naturally present in the R20291 strain. The sizes of the bands vary depending on the SLCT. Note that the SLCT-11 LMW subunit is not migrating to the expected size because it is glycosylated. M, MW marker.

**FIG 2:**
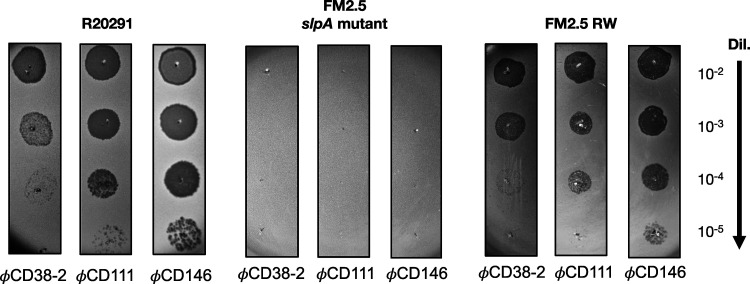
Spot test assays with WT R20291, the FM2.5 *slpA* mutant, and the FM2.5RW watermarked revertant. Serial 10-fold dilutions of phages ϕCD38-2, ϕCD111, and ϕCD146 were spotted on top of bacterial lawns of the indicated strains (titers of undiluted phage stocks = 10^9^ PFU/mL). Dark zones indicate bacterial lysis.

As a first step, glycine extracts prepared from the WT R20291 and the FM2.5 *slpA* mutant confirmed the absence of SlpA from the cell surface of the latter (Fig. 1). Then, using a spot test assay, we challenged the FM2.5 mutant with the three siphophages and used the WT R20291 strain as a control (Fig. 2, left). The absence of SlpA from the cell surface led to complete resistance to the three phages, and no phage mutants could be observed in all our assays (Fig. 2, middle). To confirm that SlpA is the receptor used by siphophages, we performed complementation assays. The introduction of a plasmid carrying a gene encoding SLCT-4 in the R20291 background (which naturally expresses SLCT-4) was previously shown to lead to homologous recombination with the chromosomal copy. Hence, we used the revertant strain FM2.5RW, in which the chromosomal mutated gene has been replaced with a “watermarked” functional *slpA* gene containing two synonymous mutations (35). The expression of SlpA in the FM2.5RW strain was confirmed by glycine extraction of cell surface proteins (Fig. 1, left, 4th lane), and spot test assays confirmed full restoration of susceptibility to phage infection (Fig. 2, right). Together, these results clearly suggest that SlpA is also a receptor for these siphophages.

### The lack of SlpA impairs phage adsorption to host cells.

**FIG 3:**
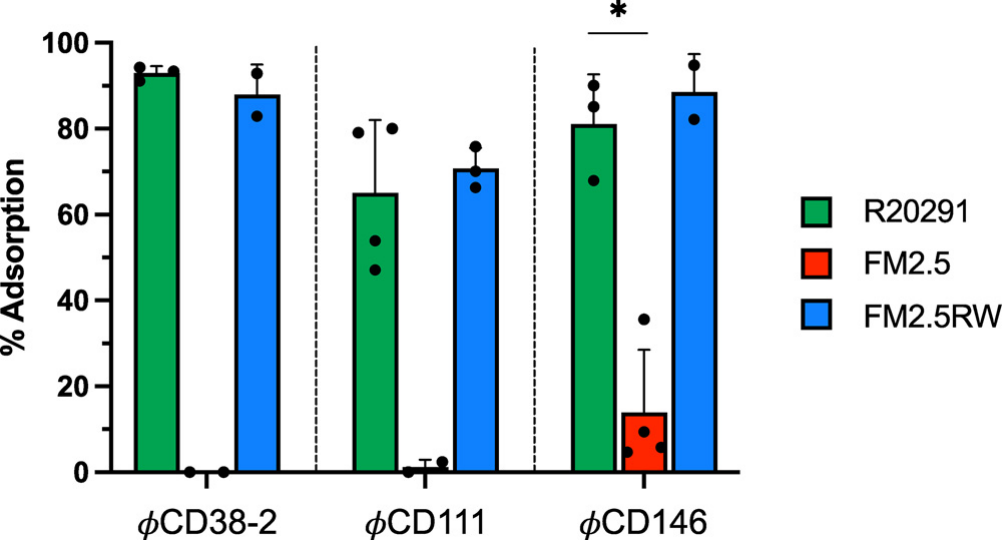
Phage adsorption assays on WT R20291, the FM2.5 *slpA* mutant, the FM2.5RW watermarked revertant, and the FM2.5 *slpA* mutant complemented with various SLCTs. Data presented are the mean values ± SEM from at least 3 technical replicates from a minimum of two independent experiments performed on different days with different cultures. *, *P* < 0.05, *t* test with Welch’s correction after logarithmic transformation of the data to reach normality based on the Shapiro-Wilk test.

The loss of phage susceptibility in the absence of SlpA suggested that phage adsorption was impaired. To verify this, we performed phage adsorption assays and compared the FM2.5 mutant with the WT R20291 strain as a control. As shown by the results in Fig. 3, the phages adsorbed to high levels onto the WT strain, with values of 92.9% ± 1.7% (mean ± standard error of the mean) for ϕCD38-2, 81.0% ± 6.7% for ϕCD146, and 65% ± 8.5% for ϕCD111. However, adsorption onto the FM2.5 mutant was severely affected, dropping to almost complete absence of adsorption for ϕCD38-2 and ϕCD111 and 13.9% ± 7.3% for ϕCD146. In our experience, adsorption values below 50% did not result in productive infection most of the time. Together, these results further supported the idea that SlpA is required by all three phages for infection, by allowing adsorption and close contact with the cell surface. Our results also suggest that in the absence of SlpA, there is no alternative receptor involved.

The three siphophages tested are genetically related and share significant DNA homology. An all-against-all BLASTn analysis using Gegenees (41) showed that ϕCD38-2 and ϕCD146 share 79% whole-genome identity, whereas ϕCD38-2 and ϕCD111 share 71% identity (data not shown). Whole-proteome comparison using Phamerator (42) confirmed the extensive similarity between the proteomes of the three phages but also revealed variations in several proteins, including the tail fiber proteins gp20/21 and the predicted receptor binding protein (RBP) gp21/22 (Fig. S1 in the supplemental material). These variations in tail fibers and RBP could possibly explain the different host ranges observed with these phages (8) and the different adsorption patterns observed on the WT strain, with ϕCD111 adsorbing less than the other two phages (Fig. 3).

### Siphophages can use more than one SLCT as their receptor.

Although the results described above strongly support the role of SlpA as a phage receptor, we could not exclude a possible destabilization of the cell surface architecture caused by the lack of SlpA, which in turn could affect phage adsorption and prevent infection. To rule out this possibility, we complemented the FM2.5 mutant with each of 12 other SLCTs described previously (Table 1) (35, 43). Of note, Avidocin-CDs have been shown to use more than one SLCT as their receptor to kill C. difficile (35). Hence, we sought to determine whether ϕCD38-2, ϕCD111, and ϕCD146 could also use alternative SLCTs to infect the R20291 strain, in addition to the natural SLCT-4.

**TABLE 1:** List of strains, plasmids, and phages used in this study

Strain, plasmid, or phage	Strain characteristics, phage genus and morphology, or plasmid description	Reference or source
Strains		
Clostridium difficile		
R20291	Epidemic isolate, ribotype 027	54
R20291 FM2.5	R20291 FM2.5 mutant carrying a G283A mutation in the *slpA* gene (SLCT-4) creating a premature stop codon (TAA) at position 289	35
R20291 FM2.5RW	R20291 FM2.5 revertant carrying a functional watermarked copy of the *slpA* gene (SLCT-4) with two synonymous changes (A282G and A285T)	35
LCUS 1039	R20291 FM2.5 *slpA* mutant containing pJAK017 expressing SLCT-1 under the control of the P_tet_ inducible promoter	This study
LCUS 1043	R20291 FM2.5 *slpA* mutant containing pJAK023 expressing SLCT-2 under the control of the P_tet_ inducible promoter	This study
LCUS 1040	R20291 FM2.5 *slpA* mutant containing pJAK018 expressing SLCT-6 under the control of the P_tet_ inducible promoter	This study
LCUS 1046	R20291 FM2.5 *slpA* mutant containing pSEW027 expressing SLCT-7 under the control of the P_tet_ inducible promoter	This study
LCUS 1041	R20291 FM2.5 *slpA* mutant containing pJAK001 expressing SLCT-7b under the control of the P_tet_ inducible promoter	This study
LCUS 1042	R20291 FM2.5 *slpA* mutant containing pJAK019 expressing SLCT-8 under the control of the P_tet_ inducible promoter	This study
LCUS 1371	R20291 FM2.5 *slpA* mutant containing pJAK019.2 expressing SLCT-8 under the control of the P_cwp2_ promoter	This study
LCUS 1047	R20291 FM2.5 *slpA* mutant containing pJAK020 expressing SLCT-9 under the control of the P_tet_ inducible promoter	This study
LCUS 1375	R20291 FM2.5 *slpA* mutant containing pJAK020.2 expressing SLCT-9 under the control of the P_cwp2_ promoter	This study
LCUS 1038	R20291 FM2.5 *slpA* mutant containing pJAK003 expressing SLCT-10 under the control of the P_tet_ inducible promoter	This study
LCUS 1048	R20291 FM2.5 *slpA* mutant containing pAAM0013 expressing SLCT-11 under the control of the P_tet_ inducible promoter	This study
LCUS 1374	R20291 FM2.5 *slpA* mutant containing pAAM0013.2 expressing SLCT-11 under the control of the P_cwp2_ promoter	This study
LCUS 1045	R20291 FM2.5 *slpA* mutant containing pJAK021 expressing SLCT-12 under the control of the P_tet_ inducible promoter	This study
LCUS 1044	R20291 FM2.5 *slpA* mutant containing pJAK022 expressing SLCT-13 under the control of the P_tet_ inducible promoter	This study
LCUS 1372	R20291 FM2.5 *slpA* mutant containing pJAK022.2 expressing SLCT-13 under the control of the P_cwp2_ promoter	This study
LCUS 1037	R20291 FM2.5 *slpA* mutant containing pJAK002 expressing SLCT-H2/6 under the control of the P_tet_ inducible promoter	This study

Escherichia coli		
TOP10	Cloning strain for pRPF plasmids	Invitrogen
CA434	HB101 carrying plasmid R702 used for conjugation into C. difficile	10
Bacteriophages		
ϕCD38-2	*Leicestervirus*; siphophage	45
ϕCD111	*Leicestervirus*; siphophage	8
ϕCD146	*Leicestervirus*; siphophage	8
ϕCD506	*Sherbrookevirus*; myophage	8
ϕCD508	*Colneyvirus*; myophage	8
ϕMMP02	*Colneyvirus*; myophage	44
ϕMMP03	*Yongloolinvirus*; myophage	8
ϕMMP04	*Sherbrookevirus*; myophage	8
Plasmids		
pRPF144	Reporter plasmid carrying the *gusA* gene under the control of the P_cwp2_ constitutive promoter and cloned into the BamHI and SacI sites.	52
pRPF185	Reporter plasmid carrying the *gusA* gene under the control of the P_tet_ inducible promoter and cloned into the BamHI and SacI sites.	52
pJAK001	pRPF185:SLCT-7b	35
pJAK002	pRPF185:SLCT-H2/6	35
pJAK003	pRPF185:SLCT-10	35
pJAK017	pRPF185:SLCT-1	35
pJAK018	pRPF185:SLCT-6	35
pJAK019	pRPF185:SLCT-8	35
pJAK019.2	pRPF144:SLCT-8	35
pJAK020	pRPF185:SLCT-9	35
pJAK020.2	pRPF144:SLCT-9	35
pJAK021	pRPF185:SLCT-12	35
pJAK022	pRPF185:SLCT-13	35
pJAK022.2	pRPF144:SLCT-13	35
pJAK023	pRPF185:SLCT-2	35
pSEW027	pRPF185:SLCT-7	35
pAAM0013	pRPF185:SLCT-11	35
pAAM0013.2	pRPF144:SLCT-11	35

Representative members from each of the 12 SLCTs, cloned into the pRPF185 plasmid backbone under the control of a P_tet_ tetracycline-inducible promoter or into the pRPF144 plasmid under the control of the constitutive promoter P_cwp2_, were transferred by conjugation and expressed in the FM2.5 mutant. Correct expression of each SLCT upon anhydrotetracycline induction was confirmed by SDS-PAGE after glycine extraction (Fig. 1). The levels of expression of the SLCTs cloned in front of the constitutive P_cwp2_ promoter were comparable to the levels of expression of the induced forms (Fig. S2).

**FIG 4:**
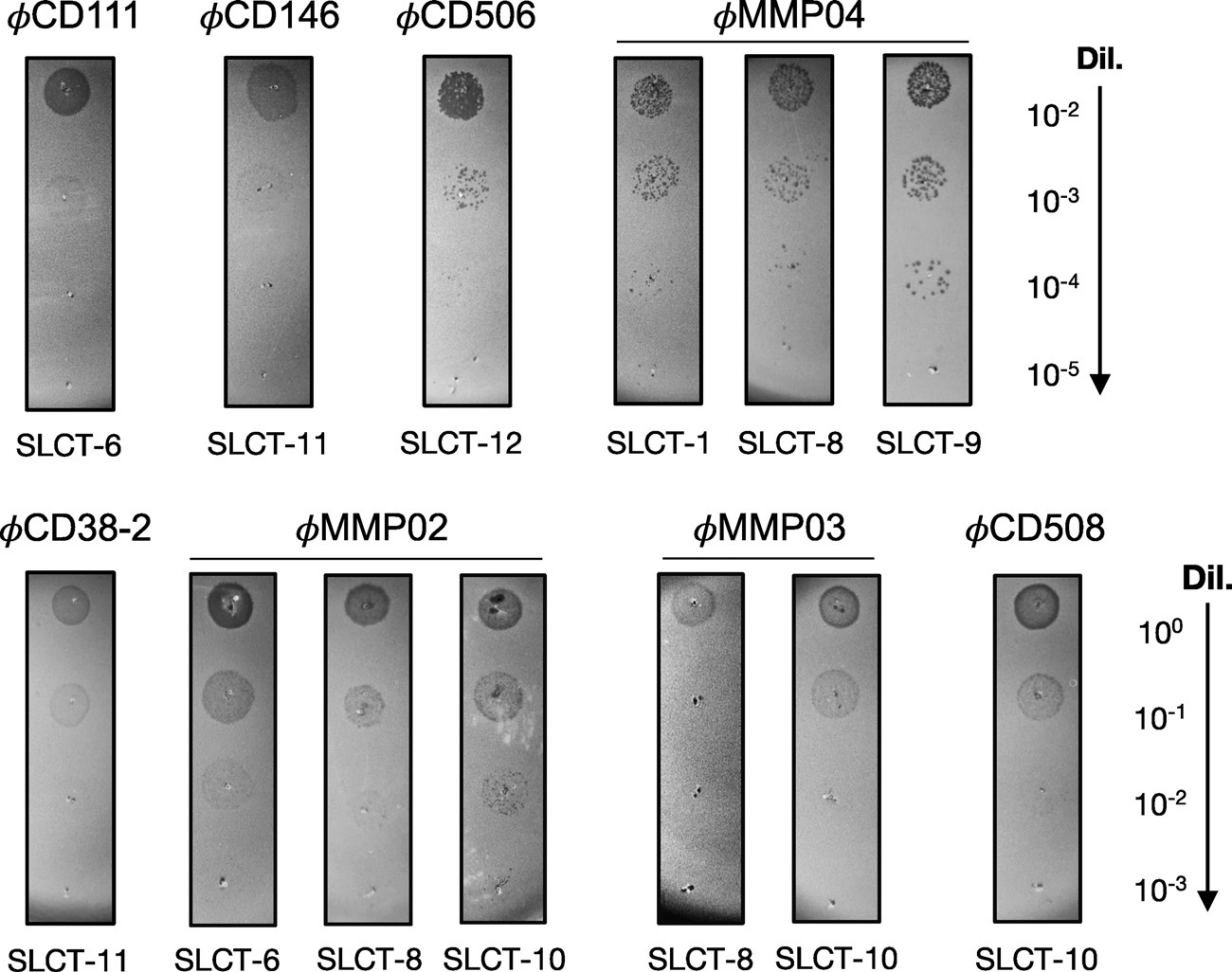
Phage susceptibility assays of the FM2.5 *slpA* mutant complemented with different SLCTs. Serial 10-fold dilutions of the indicated phages (titers of undiluted phage stocks = 10^9^ PFU/mL) were spotted on top of bacterial lawns of the indicated strains. Darker zones/plaques indicate bacterial lysis, although extensive lysogeny was observed with some phages, making spots more difficult to observe. The experiments were repeated several times, and only representative positive infections are shown here. Also, nonspecific lysis was not observed in the absence of a productive infection, since no lysis zone could be seen with other SLCTs using undiluted phage lysates.

Next, phage susceptibility assays were performed with all of the complemented strains and the three siphophages. The expression of SLCT-6 restored susceptibility to ϕCD111, and SLCT-11 rendered bacteria susceptible to infection by ϕCD38-2 and ϕCD146 (Fig. 4). All of the other SLCT-complemented strains were found to be resistant to infection by the siphophages, and no lysis zones were detected (data not shown and Table 2). The predicted RBPs (gp21) from ϕCD38-2 and ϕCD146 are 100% identical, while they share 82% identity with gp22 from ϕCD111 (Fig. S1B). This might explain why ϕCD111 did not infect cells expressing SLCT-11 and why only ϕCD111 infected cells expressing SLCT-6. We cannot exclude that other tail proteins could be involved in receptor recognition as well. For instance, in the present case, the tail fiber protein gp20 from ϕCD38-2 is different from gp20 from ϕCD146 and from gp21 of ϕCD111. These differences could possibly explain our observations, although further investigation will be required (Fig. S1C). Nevertheless, our results show that the three siphophages tested can use at least two SLCTs each to infect C. difficile and that this interaction is specific, since not all SLCTs could restore susceptibility to infection.

**TABLE 2:** Susceptibility to phage infection of the FM2.5 mutant complemented with various SLCTs

Strain	SLCT	% adsorption ± SEM of^*a*^:							
		Siphophage:			Myophage:				
		ϕCD38-2	ϕCD111	ϕCD146	ϕCD506	ϕCD508	ϕMMP02	ϕMMP03	ϕMMP04
R20291	4	92.9 ± 1.7	65 ± 8.5	81 ± 6.7	1.5 ± 12.8	0 ± 12.8	16	0	0
FM2.5	No SlpA	0 ± 0.9	1.2 ± 1.2	13.9 ± 7.3	11.8 ± 4.5	8.8 ± 6.3	7.6 ± 12.2	0 ± 13.5	0 ± 12.8
FM2.5RW	4^*b*^	87.9 ± 5	70.7 ± 2.8	88.5 ± 6.3					
LCUS 1039	1								ND
LCUS 1043	2								
LCUS 1040	6		ND				ND		
LCUS 1046	7								
LCUS 1041	7b								
LCUS 1371	8^*c*^					11.3 ± 0.2	85.3 ± 4.1	53.1 ± 8	96.8 ± 0.3
LCUS 1375	9^*c*^								98.9 ± 0.2
LCUS 1378	10				3.4 ± 7.9	87.7 ± 0.4	74.8 ± 6.1	86.7 ± 5.1	0 ± 0.3
LCUS 1374	11^*c*^	97.5 ± 0.7	0	98.5 ± 0.5					
LCUS 1045	12				54 ± 5.4	15.6 ± 6.1			
LCUS 1372	13^*c*^								
LCUS 1037	H2/6								

a Shading indicates positive infection; empty cells indicate nonsusceptibility; ND indicates that % adsorption was not determined. The genera of the phages are listed in Table 1.

b Watermarked chromosomal *slpA* gene.

c The expression of this SLCT was under the control of the constitutive Pcwp2 promoter.

### Expression of specific SLCTs in the FM2.5 mutant confers susceptibility to additional phages.

Besides the siphophages ϕCD38-2, ϕCD111, and ϕCD146, no other phages from our collection could form plaques on the WT R20291. The most likely reason to explain the lack of infection by some phages is the absence of a suitable host receptor on the bacterial surface.

Different myophages from our collection were used in spot test assays against the FM2.5 mutant carrying one of the 12 SLCTs expressed from a plasmid. As shown by the results in Table 2 and Fig. 4, the complemented mutant became susceptible to infection by ϕCD506, ϕCD508, ϕMMP02, ϕMMP03, and ϕMMP04 when SLCT-1, -6, -8, -9, 10, or -12 was expressed. These results confirm that SlpA is also used as a receptor by several myophages, thus corroborating previous studies (35, 38, 39). Of note, three of the myophages also recognized more than one SLCT. Also, except for ϕMMP02 and ϕCD111, which both recognized SLCT-6, myophages and siphophages seemed to bind different SLCTs, and no overlap was observed in susceptibility testing. We performed phage adsorption assays to investigate the attachment of the phages depending on the SLCT expressed. As shown by the results in Table 2, high adsorption ratios were observed for phages that were able to infect a given complemented strain, but little to no adsorption was observed when no infection occurred. Although we did not test adsorption with all possible combinations of phages and SLCTs, a minimum of ~50% adsorption was found to be necessary to allow infection in our assays, while little to no adsorption correlated with the absence of infection.

The phages used in this study were originally isolated on four different strains (8, 44, 45). Strain LCUS0274, a ribotype 027 strain on which ϕCD38-2, ϕCD111, and ϕCD146 were isolated and propagated, expresses SLCT-4, like the R20291 strain in this study. Likewise, phages ϕCD508, ϕMMP02, and ϕMMP03 were isolated on strain LCUS0117 (8, 44), which expresses SLCT-10. Accordingly, these phages recognized SLCT-10 in the FM2.5 complemented strain (Table 2). In summary, different myophages can use one or more SLCTs as their receptor. These results clearly demonstrate that SlpA is a general receptor used by many C. difficile phages.

### The D2 domain of the LMW fragment of SLCT-4 is essential for adsorption and infection by ϕCD38-2 and ϕCD146.

**FIG 5:**
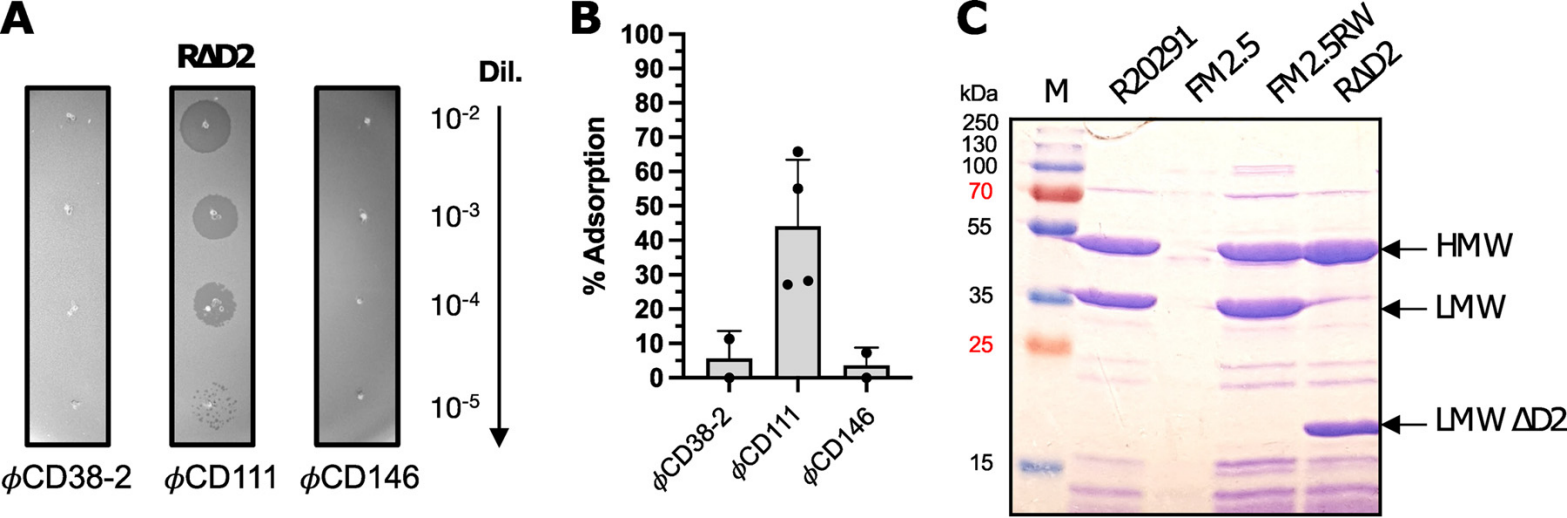
Susceptibility to phage infection of the RΔD2 mutant lacking domain D2 of the LMW fragment of SLCT-4. (A) Five-microliter amounts of serial dilutions (10^−2^ to 10^−5^) of phages ϕCD38-2, ϕCD111, and ϕCD146 (initial lysates at 10^9^ PFU/mL) were spotted onto a bacterial lawn of the RΔD2 mutant. Dark zones indicate productive infection and bacterial lysis. (B) Phage adsorption assays on the RΔD2 mutant. Data presented are the mean values ± SEM from at least 3 technical replicates from a minimum of two independent experiments. (C) Coomassie-stained 12% SDS-PAGE gel showing glycine extractions of surface proteins from WT R20291, the FM2.5 *slpA* mutant, the FM2.5RW watermarked revertant, and the RΔD2 mutant. The arrows indicate the major bands corresponding to the HMW, LMW, and LMW ΔD2 units of the SLCT-4 naturally present in the R20291 strain. M, MW marker.

In order to identify potential subdomains of the SlpA protein that could be involved in the interaction with phages, we conducted phage infection assays and adsorption tests using the recently described RΔD2 mutant strain. This strain was created by replacing the chromosomal copy of SLCT-4 in the R20291 strain with a copy lacking 145 amino acids corresponding to the D2 domain within the LMW fragment (27). Even though nearly half of the LMW fragment is missing in the RΔD2 strain, an S-layer with lattice characteristics identical to those of the wild-type strain is formed, except that the outermost staggered ridge feature is absent in the mutant. Infection assays with the 3 siphophages showed that only ϕCD111 was able to infect the RΔD2 mutant, whereas ϕCD38-2 and ϕCD146 could not infect this strain (Fig. 5). Adsorption tests showed that the binding of ϕCD111 onto the mutant was lower than on the wild-type strain (44% ± 19% versus 65% ± 8.5%, respectively), but this phage was still able to form plaques with similar efficacy. On the contrary, the adsorption of ϕCD38-2 and ϕCD146 was almost completely abolished (5.7% ± 7.8% and 3.7% ± 5.2%, respectively), and plaques did not form. These results strongly suggest that the D2 domain of SlpA is essential for binding and infection by ϕCD38-2 and ϕCD146 but is dispensable for ϕCD111. Whether the D2 domain is also essential for binding of other myophages will need to be determined in future studies.

### Relationship between SLCT, phage RBP, and susceptibility to infection.

**FIG 6:**
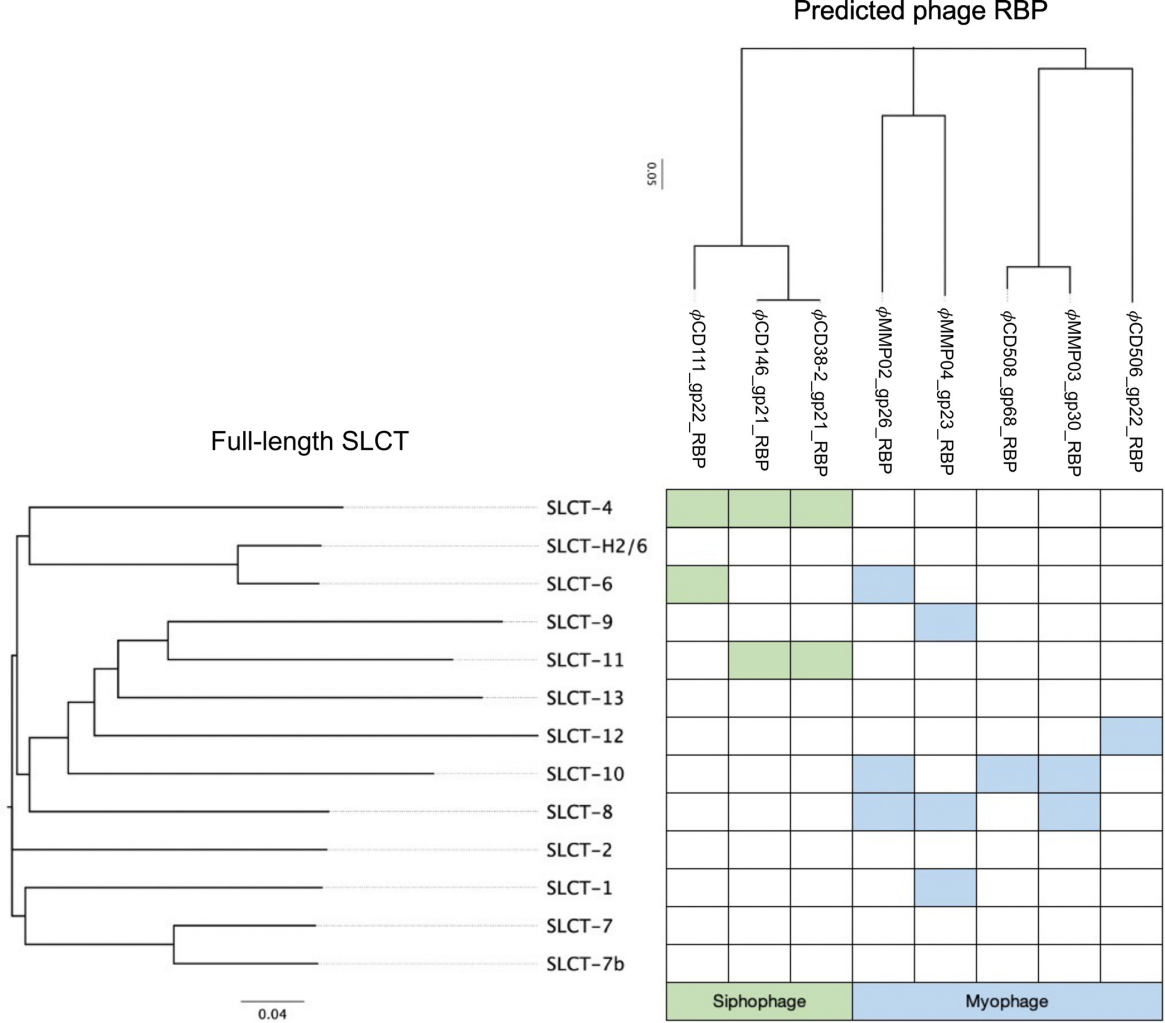
Susceptibility to phage infection as a function of SLCT and RBP phylogeny. The full-length SlpA amino acid sequences were aligned, and a phylogenetic tree was constructed, creating 4 different clades (indicated by colored lines). The amino acid sequences of the predicted phage RBPs were also aligned, and a phylogenetic tree was built. The branches of both trees were reorganized as a function of the susceptibility to phage infection (shaded cells).

We sought to determine if some relationship could be made between the SLCT, the phage RBP, and the observed susceptibility to infection. Multiple alignments were built using the SlpA protein on one hand and the predicted phage RBPs on the other (Fig. S3 and S4). Phylogenetic trees were generated and then compared with the susceptibility profiles of the different complemented strains (Fig. 6). Although some of the RBPs are highly similar (e.g., ϕCD146 versus ϕCD111 or ϕCD508 versus ϕMMP03), slight differences in host range were observed. This suggests that some of the divergent regions between these RBPs are involved in binding with these specific SlpA isoforms or that other phage proteins are involved in binding, such as the tail fiber gp20/21 (Fig. S1). Regarding the myophages, ϕMMP02 and ϕMMP04 both recognized SLCT-8, and although they were grouped together in the phylogenetic tree based on their RBPs, the level of amino acid conservation is only 67% over 38% of the protein. On the other hand, ϕCD508 and ϕMMP03 recognized SLCT-10 and their RBPs are closely related and share 92% amino acid identity over 100% of the protein, and yet, SLCT-8 is not bound by ϕCD508, whereas it is by ϕMMP03. In addition, ϕMMP02 and ϕMMP03 both recognized SLCT-8 and SLCT-10, but they share only 23% sequence identity over 50% of the protein. These observations make prediction of the phage host range based on the RBP alone very difficult. Note that we also did a clustering analysis using only the LMW fragments of the SLCTs to generate the phylogenetic tree and very similar results were obtained (Fig. S4 and S5). In summary, although we could observe tendencies, larger analyses will be necessary to draw conclusions linking a particular RBP with specific SLCTs.

## DISCUSSION

The identification of the receptor(s) used by phages to infect C. difficile has been a topic of great interest for many years. Here, we used a panel of 3 siphophages and 5 myophages in a single bacterial model based on the FM2.5 *slpA* mutant isolated previously (35) to provide experimental evidence that many phages of C. difficile use the cell surface protein SlpA as their receptor to initiate infection. Previous work on Avidocin-CDs provided indirect evidence that C. difficile myophages could use SlpA as their host receptor (35). More recent reports brought additional evidence that the S-layer acts as the receptor for two C. difficile myophages (ϕCD1801 and ϕHN10) (37, 38) and a siphophage (CDHS-1) (39). One study provided direct evidence that SlpA, and more specifically the LMW fragment, is the receptor used by ϕHN10 (40). The report by Whittle et al. involved adsorption assays with the myophage ϕCD1801 and C. difficile strain 630 coexpressing one of three different SLCTs in addition to its native S-layer (38). However, expression of the exogenous SlpA gene was not confirmed and, therefore, the authors could not completely exclude the possibility that an indirect effect could be responsible for the observed adsorption results. Also, the absence of infection in the strain 630 background did not allow the conclusion that SlpA could be used by phages as a receptor to infect cells and that adsorption was not due to other factors. Finally, the recent report on the siphophage CDHS-1 showed that the absence of an S-layer in the R20291 FM2.5 mutant prevented phage infection, suggesting that SlpA is the receptor used by this phage. However, in the absence of complementation and adsorption assays, it could not be fully concluded that SlpA was really the receptor used by CDHS-1 (39).

Because all our experiments were conducted in the same genetic background and SlpA isoforms were expressed only one at a time in complementation assays, we could readily compare the infection efficacies of our phages as a function of the SLCT expressed. While this approach alleviated several of the limitations that occur during phage host range assays using multiple bacterial strains, the level of expression of SlpA from a plasmid was generally lower than from the normal chromosomal allele. Except for SLCT-8, which was expressed to levels close to the natural SLCT-4, all the other SLCTs were expressed to lower levels, even when using the constitutive promoter P_cwp2_. Consequently, many of the positive infections observed with some SLCTs were weaker than what is normally observed with the natural host of the corresponding phages. Nevertheless, we can exclude a problem in the expression of SlpA since adsorption was high in most cases of positive infection and adsorption values were very similar to those observed on the natural replicating host of the corresponding phages. For example, ϕCD38-2 and ϕCD146 adsorbed to very high levels on SLCT-11 but infected these cells less efficiently than the WT R20291. Also, we could see strong infection with ϕMMP04 on SLCT-1, -8, and -9, even though SLCT-1 was not expressed as efficiently as the other two SLCTs. A previous study by Thanki et al. (46) suggested that high adsorption (>80%) was required for productive infection; however, we could still detect productive infection with certain phages even when their adsorption was only slightly above 50% (e.g., ϕMMP03 on SLCT-8 and ϕCD506 on SLCT-12). In addition, using the RΔD2 mutant, the adsorption of ϕCD111 was even lower, with values slightly below 30% in some experiments, and still, this phage was able to form plaques with the same efficacy. Therefore, the differences observed in the efficacy of infection might be related to factors other than adsorption *per se*. Supporting this, our three siphophages can infect the WT R20291 strain very well, so we can rule out the possibility that the reduced efficacy of infection observed with ϕCD38-2 on SLCT-11 was due to problems in replication of the phages in this strain. One possible explanation could be that the interaction between the RBP and different SLCTs is not always optimal despite strong adsorption. The process of baseplate docking to the receptor might also be compromised. Conformational changes in the baseplate structure, as seen in the lactococcal phage p2 (19, 20), might also be required for infection but compromised with certain SLCTs.

It is worthy of mention that 8 of the 13 SLCTs tested could be targeted by phages in our study. The absence of infection with 5 SLCTs could simply reflect the small phage collection used herein. Nevertheless, an interesting observation we made was that the SLCTs recognized by siphophages and myophages were different, except for SLCT-6, suggesting that the two phage morphologies have different binding patterns. Also, SLCT-8 and SLCT-10 were recognized by multiple myophages with diverse RBPs. These SlpA isoforms are not closely related, so it will be interesting to investigate whether specific domains, structures, or amino acid residues could explain this greater binding capacity. Our clustering analysis did not allow us to find any clear link between SLCTs, RBPs, and susceptibility to infection. Similar conclusions were reported whereby phages with identical tail fibers displayed different host ranges (46). Along the same line, our experiments with the RΔD2 mutant revealed how closely related phages like ϕCD111, ϕCD38-2, and ϕCD146 could be affected differently by deletion of the outermost portion of the LMW fragment. Our data suggest that the D2 domain is involved in adsorption of these phages, but not of ϕCD111. The latter phage possibly interacts with another region of the LMW fragment (e.g., the D1 domain) or with the HMW fragment, or both. Further experiments will be required to determine which region of SlpA is interacting with ϕCD111. All these elements argue in favor of a complex interaction that would involve the RBP and, possibly, additional tail components. Future studies with a more diverse panel of well-characterized phages and SlpA constructions will be required to get a refined view of the interaction between phages and the S-layer.

Phage therapy has gained interest in recent years, and treatment of C. difficile infections with therapeutic phages could have huge advantages over currently available antibiotics. Therefore, phage therapy has been viewed as a potential alternative to fight C. difficile infections (3, 4). However, it is currently constrained by the fact that all phages known to infect C. difficile are temperate, and thus, capable of lysogeny (7). Using these phages for therapy is not advisable, as exemplified by several reports showing growth rebound and lysogeny after treatment, particularly when single phages were used (7, 47–50). Fortunately, the issue of lysogeny can be circumvented by genetic engineering (50), but another important limitation is the relatively narrow host range of available phages. Even with phage cocktails, it is difficult to target multiple strains (3). Here, experimental demonstration that SlpA is a general host receptor used by many C. difficile phages opens the way to genetic engineering of tail genes to target multiple strains with a limited number of phages. One could argue that mutation of the SlpA protein would quickly lead to phage resistance, which is true, and the FM2.5 and FM2.6 mutants confirm this possibility. However, it was shown that the lack of an S-layer comes with a huge fitness and virulence cost (35). Therefore, the development of phage resistance through SlpA mutation would be deleterious for phage therapy applications but would also strongly impair C. difficile’s capacity to cause disease.

In summary, our study brings additional experimental proof that SlpA is a receptor used by both myophages and siphophages and, therefore, seems to be a general phage receptor. Much remains to be done to fully understand how C. difficile phages interact with the S-layer, including how the RBP binds to SlpA and whether accessory proteins are involved or if the adsorption and/or DNA injection mechanisms varies between phage subgroups. Ongoing structural work that aims at reconstructing phage architecture will clearly bring insightful knowledge in this regard.

## MATERIALS AND METHODS

### Bacterial strains, plasmids, and bacteriophages.

A comprehensive list of bacterial strains, plasmids, and phages used in this work is presented in Table 1. C. difficile was grown in an anaerobic chamber (anaerobic conditions of H_2_ 10%, CO_2_ 5%, and N_2_ 85%; Coy Laboratories) at 37°C in prereduced brain heart infusion (BHI) broth or TY broth (2% yeast extract, 3% tryptose, pH 7.4). Thiamphenicol (15 μg mL) or norfloxacin (12 μg/mL) was added when necessary. Escherichia coli was grown under aerobic conditions in Luria-Bertani (LB) broth in an incubator with agitation at 37°C, with chloramphenicol (25 μg/mL) or kanamycin (50 μg/mL) when necessary.

### Bacteriophage amplification and titration.

Phage lysates were prepared in TY broth using standard phage amplification protocols, and titers were determined using the soft agar overlay method, as described previously (51). Phage lysates were filtered through 0.45-μm membranes and stored at 4°C. Phage titers were verified regularly, and stocks contained >10^9^ PFU/mL.

### Conjugation of plasmid DNA into C. difficile.

Plasmids carrying the different SLCTs described previously were transferred by conjugation into the FM2.5 mutant strain as previously described (Table 1) (11). All manipulations were performed under an anaerobic atmosphere using prereduced medium and buffer. Briefly, the different Escherichia coli CA434 donor strains containing SlpA plasmids were grown overnight in LB broth containing 50 μg/mL kanamycin and 25 μg/mL chloramphenicol. The C. difficile FM2.5 receptor strain was grown in TY broth overnight. A 3% inoculum was then used to inoculate BHIS (brain heart infusion supplemented with 5 g/L yeast extract) broth. The optical density at 600 nm (OD_600_) was monitored until it reached 0.5. The E. coli donor strain was centrifuged at 4,000 × *g* for 2 min and gently resuspended in 1× phosphate-buffered saline (PBS). The donor strain was spun down again, and the supernatant was discarded. Next, 200 μL of the C. difficile cell suspension was used to gently resuspend the donor strain under anaerobic conditions. The resulting coculture was then spotted onto prereduced BHIS plates and incubated for 8 to 24 h. The cells were then collected using 1 mL 1× PBS, followed by centrifugation. The supernatant was discarded, and the cells were resuspended in 500 μL of BHIS broth and then plated on BHIS plates containing 15 μg/mL thiamphenicol and 12 μg/mL norfloxacin. The plates were incubated at 37°C for up to 72 h. Transconjugants were restreaked on TY plates containing 15 μg/mL thiamphenicol and 12 μg/mL norfloxacin, incubated for 24 h at 37°C, and verified by PCR for the presence of the plasmid using SLCT-specific primers.

### Induction of *slpA* expression in C. difficile.

The plasmids containing genes encoding each of the 12 SlpA isoforms cloned into the pRPF185 plasmid were under the control of the inducible P_tet_ promoter (35, 52). To induce the expression of SlpA, bacteria were grown in 10 mL of yeast extract-tryptose (TY) broth up to an OD_600_ of 0.4 and anhydrotetracycline was added to a final concentration of 20 ng/mL. Cultures were grown overnight and used to assess susceptibility to phage infection and the level of SlpA expression by SDS-PAGE following glycine extraction (see below).

### Subcloning of SLCTs for constitutive expression in C. difficile.

SLCT-8, -9, -11, and -13 were subcloned into the pRPF144 plasmid to allow constitutive expression of the *slpA* gene under the control of the P_cwp2_ promoter. To do this, the pJAK019, pJAK020, pJAK022, and pAAM013 plasmids (Table 1) were digested with BamHI and SacI restriction enzymes to excise the *slpA* gene. The pRPF144 plasmid was also digested with BamHI and SacI to remove the *gusA* gene (52). Digested fragments were extracted from a 0.8% agarose gel using the QIAquick gel extraction kit (Qiagen, Mississauga, ON). The *slpA* gene fragments were then ligated into the pRPF144 backbone using T4 DNA ligase at room temperature for 1 h, and the ligation products were transformed in E. coli MC1061 using standard protocols. The resulting plasmids were purified using the GeneAid high-speed minikit (FroggaBio, Concord, ON) and then sequenced at the Université Laval sequencing center. The validated plasmids were then transformed into E. coli CA434 donor cells and conjugated into the C. difficile FM2.5 strain as described above.

### Glycine extraction of cell surface proteins.

We assessed the expression of the SlpA proteins by SDS-PAGE after performing a surface protein extraction of the induced cultures. The samples were prepared as follows: 10-mL amounts of the induced C. difficile cultures were centrifuged at 4,000 × *g*, followed by a washing step with 1× PBS. We suspended the pellet with 200 μL of 0.2 M glycine, pH 2.2, followed by incubation at room temperature for 30 min. We centrifuged again during 5 min at 10,000 × *g*. Then, we transferred 150 μL of the supernatant into a new tube and adjusted the pH to 7.5 using a solution of 2 M Tris-HCl. We mixed 15 μL of the samples and 5 μL of 4× loading buffer (200 mM Tris-HCl, pH 6.8, 400 mM dithiothreitol [DTT], 8% SDS, 0.4% bromophenol blue, and 40% glycerol). We then ran the samples on 12% polyacrylamide gels (BioShop). The migration of the sample was performed in a Mini-Protean tetra cell apparatus (Bio-Rad, Mississauga, ON, Canada), using a voltage of 100 V for 25 min, followed by 1 h at 150 V. The gels were stained with Coomassie blue.

### Phage susceptibility testing on FM2.5 SLCT-complemented strains.

Spot tests using a standard soft agar overlay method were used to determine the susceptibility to phage infection of different FM2.5 complemented strains (51). The night before the experiment, a preculture of a complemented strain was inoculated into 5 mL of TY broth supplemented with thiamphenicol and incubated at 37°C under anaerobic conditions. The next day, a fresh 5- to 10-mL culture was inoculated with 5% of the overnight preculture, and thiamphenicol was added, as well as 20 ng/mL anhydrotetracycline. The OD_600_ was then monitored regularly. Simultaneously, we prepared TY with 0.3% agarose maintained at a temperature of 55°C. When the bacterial culture reached an OD_600_ of 0.8, 4 mL of soft agarose was mixed with 0.67 mL of the bacterial culture and salts (100 mM MgCl_2_ and 0.3mM CaCl_2_) and with antibiotics when necessary (15 μg/mL thiamphenicol and 20 ng/mL anhydrotetracycline). We then poured the tube contents over square petri dishes containing TY bottom agar (1% agar), with antibiotics when required (15 μg/mL thiamphenicol and 20 ng/mL anhydrotetracycline). Once the top agarose had hardened, 5-μL amounts of serially diluted phage lysates (initial stocks adjusted at 10^9^ PFU/mL) were deposited directly on top of the soft agarose overlay. We incubated the petri dishes overnight at 37°C in an anaerobic chamber. Zones of lysis in the bacterial lawns revealed productive phage infections.

### Bacteriophage adsorption assays.

Phage adsorption assays were performed as described previously (11). Briefly, bacteria from an overnight culture were grown in TY broth for 24 h. Then, 0.9 mL of culture was mixed with 1 × 10^5^ PFU of the desired phage in the presence of salts (10 mM [each] MgCl_2_ and CaCl_2_) in a final volume of 1 mL. Phages were allowed to adsorb for 30 min at 37°C. Cells were then collected by centrifugation at 13,000 × *g* for 1 min. Free phages in the supernatant that did not adsorb were enumerated after serial 10-fold dilutions on standard soft agarose overlays as described earlier, and titers were analyzed against the initial phage input. The adsorption ratio was calculated using the following formula: 100 – [(residual titer/initial titer) × 100]. Each experiment was done in technical replicate. Plots were generated using the mean adsorption values ± standard errors of the means (SEM).

### Phage genome comparisons and sequence alignments.

Whole-phage-genome alignments and maps were created using Phamerator (42) with default options (53). Gene products with similar functions were grouped into phamilies (identity threshold of ≥32.5% with an E value of ≤10^−50^). Pairwise DNA alignment of the genomes was also done with Phamerator, with DNA similarity determined using BLASTn and a threshold value of 10^−4^. SlpA and RBP sequences were aligned, and phylogenetic trees were generated using the CLC Sequence Viewer 8.0 desktop application (Qiagen) with default parameters. The RBPs from myophages were predicted by performing a local BLASTp protein alignment using phage tail proteins that were suspected to be RBPs as query sequences and a local database composed of 12 RBP sequences from diffocin-4/Avidocin-CD described previously (35). The RBPs from siphophages were predicted based on genome synteny and the predicted RBP from CDHS-1, a siphophage highly similar to ϕCD38-2 (39).

### Statistical analysis.

Statistical analysis of adsorption results was done to compare groups containing at least 3 data points. A Shapiro-Wilk test for normality was done first, and if data were normally distributed, a parametric *t* test with Welch’s correction was performed. If data were not distributed normally, a Mann-Whitney nonparametric test was done or logarithmic transformation of the data was performed to reach normality, and then a *t* test with Welch’s correction was done. Statistical significance was reached if the *P* value was ≤0.05.

### Data availability.

All relevant data and source material are available from the corresponding author upon reasonable request.
